# The Impact of Systematic Laparoscopic Skills and Suture Training on Laparoscopic Hysterectomy Outcomes in a Brazilian Teaching Hospital

**DOI:** 10.1055/s-0039-1700587

**Published:** 2019-12

**Authors:** Anna Luiza Lobão Gonçalves, Helizabet Abdala Ayroza-Ribeiro, Raquel Ferreira Lima, Aline Estefane Eras Yonamine, Fabio Ohara, Paulo Augusto Galvão Ayroza-Ribeiro

**Affiliations:** 1 Gynecological Endoscopy and Endometriosis Sector, Department of Obstetrics and Gynecology, Faculdade de Ciências Médicas, Santa Casa de São Paulo, São Paulo, SP, Brazil

**Keywords:** hysterectomy, laparoscopic surgery, education, suture training, histerectomia, cirurgia laparoscópia, educação, treinamento de sutura

## Abstract

**Objective** To evaluate the impact of systematic laparoscopic skills and suture training (SLSST) on the total laparoscopic hysterectomy intra- and postoperative outcomes in a Brazilian teaching hospital.

**Methods** A cross-sectional observational study in which 244 charts of total laparoscopic hysterectomy (TLH) patients operated from 2008 to 2014 were reviewed. Patient-specific (age, parity, previous cesarean sections, abdominal surgeries and endometriosis) and surgery-related variables (hospital stay, operative time, uterine volume and operative complications) were analyzed in three different time-frame groups: 2008-09 (I-1) – TLHs performed by senior attending physicians; 2010-11 (I-2) – TLHs performed by residents before the implementation of the SLSST program; and 2012-14 (I-3) – TLHs performed by residents after the implementation of the SLSST program.

**Results** A total of 244 TLH patients (mean age: 45.93 years) were included: 24 (I-1), 55 (I-2), and 165 (I-3). The main indication for TLH was uterine myoma (66.4%). Group I-3 presented a decrease in surgical time compared to group I-2 (*p* = 0.010). Hospital stay longer than 2 days decreased in group I-3 compared to group I-2 (*p* = 0.010). Although we observed decreased uterine volume (154.2 cm^3^) in group I-2 compared to group I-1 (217.8 cm^3^) (*p* = 0.030), logistic regression did not find any association between uterine volume and surgical time (*p* = 0.103).

**Conclusion** The total operative time for laparoscopic hysterectomy was significantly shorter in the group of patients (I-3) operated after the systematic laparoscopic skills and suture training was introduced in our hospital.

## Resumo

**Objetivo** Avaliar o impacto do treinamento laparoscópico sistematizado de habilidades e sutura (TLSHS) nos resultados intra e pós-operatórios da histerectomia laparoscópica em um hospital universitário brasileiro.

**Métodos** Estudo observacional transversal de revisão de 244 prontuários de pacientes submetidas a histerectomia total laparoscópica (HTL) operadas entre 2008 e 2014. Os fatores específicos das pacientes (idade, paridade, cesariana prévia, cirurgias abdominais, e endometriose) e as variáveis relacionadas à cirurgia (tempo de hospitalização, tempo de cirurgia, volume uterino e complicações operatórias) foram analisados em três grupos temporais: 2008-09 (I-1) – HTLs realizadas por médicos experientes; 2010-11 (I-2) – HTLs realizadas por residentes sem TLSHS; 2012-2014 (I-3) – HTLs realizadas por residentes após a implementação do TLSHS.

**Resultados** Um total de 244 pacientes submetidas a HTLs foram incluídas (média de idade de 45,93 anos): 24 operadas no período I1, 55 no I2, e 165 no I3. A principal indicação para HTL foi mioma uterino (66,4%). O grupo I-3 apresentou diminuição no tempo cirúrgico quando comparado ao grupo I-2 (*p* = 0,010). Hospitalização superior a 2 dias diminuiu no grupo I-3 comparado ao grupo I-2 (*p* = 0,010). Apesar de observarmos diminuição no volume uterino dos pacientes do grupo I-2 (154,2 cm^3^) em comparação com os do grupo I-1 (217,8 cm^3^) (*p* = 0,030), a regressão logística não revelou associação entre volume uterino e tempo cirúrgico (*p* = 0,103).

**Conclusão** O tempo cirúrgico na HTL foi significativamente menor no grupo de pacientes submetidas à cirurgia após a implantação do TLSHS em nosso hospital.

## Introduction

Laparoscopy-assisted hysterectomy has evolved as an alternative to conventional open surgery since the end of the twentieth century.^1^ It uses cameras and specific instruments to remove the uterus, the fallopian tubes, and/or the ovaries through a minimally-invasive trans-vaginal access.^2^ ^3^ The procedure is called total laparoscopic hysterectomy (TLH) when hemostatic clamping of the uterine vessels, resection of the uterosacral and cardinal ligaments, and colporrhaphy are all performed through a minimally-invasive video-assisted approach.^3^ The benefits of TLH are diminished postoperative pain, lower use of opioid analgesics, shorter hospital stay, early rehabilitation and return to work, minimal blood loss, enhanced visualization of intra-abdominal structures, which minimizes the risk of iatrogenic lesion to bladder and ureters, and lower rate of short- and long-term complications.^4^ ^5^ ^6^ Additonally, the intrafascial dissection technique preserves the vaginal apex support structures, maintaining vaginal length.^6^ ^7^ ^8^ ^9^

However, TLH is not widespread in many countries.^5^ ^7^ ^10^ ^11^ ^12^ Data from the Brazilian Unified Healthcare System reveals only 2,947 laparoscopic procedures out of 932,382 hysterectomies performed from 2008 to 2017.^13^ Developed countries like the United States and England estimate that 20% to 30% of hysterectomies are laparoscopic-assisted.^10^ ^14^ The major struggle regarding laparoscopic surgery expansion has been to train new surgeons. The long learning curve to achieve proficiency in two-dimension screen vision, camera navigation, hand-eye coordination, and psychomotor skills to handle laparoscopic tools with dexterity conflict with a limited number of procedures and professionals in teaching hospitals with scarce resources.^7^ ^12^ ^15^ ^16^ On the other hand, increased demand for laparoscopic procedures in private health systems pressure junior surgeons to take up complex cases they may not be proficient to deal with, resulting in lower surgical performances and increased morbidity and mortality.^15^ ^17^

Laparoscopic psychomotor skills must be preliminarily acquired by practicing on specific validated training models outside the operating room.^15^ ^16^ ^17^ Similar to a video game, training platforms enable the repetitive practice of standard laparoscopic tasks. They also evaluate performance objectively, and provide feedback to the trainees.^18^ Simulators were proven to shorten surgical time and improve perioperative morbidity in TLH procedures,^19^ and they potentially reduce the learning curve compared to traditional surgical teaching methods.^20^ Current surgical practice regulations demand a controlled, standardized and validated training program for new laparoscopic surgeons, such as the “Winners” program in Europe and the American College of Obstetricians and Gynecologists (ACOG) Fundamentals of Laparoscopic Surgery (FLS) program, in the US.^15^ In an effort to validate an implemented standardized laparoscopic training in Brazil, the present study evaluated the impact of the systematic laparoscopic skill and suture training (SLSST) on the outcomes of TLH performed in a teaching hospital (Santa Casa de Misericórdia de São Paulo, in the city of São Paulo, Brazil) from 2008 to 2014. We hypothesized that the SLSST would have a positive impact on the intra- and postoperative outcomes of TLH.

## Methods

The present research was approved by the Ethics in Human Research Committee and Institutional Review Board of Santa Casa de Misericórdia de São Paulo (under number: 14945313.8.0000.5479)

We conducted a cross-sectional observational study in which 610 charts of patients submitted to hysterectomy at Santa Casa de Misericórdia de Sãoo Paulo from 2008 to 2014 were reviewed. All TLHs were included in the study, corresponding to 40% (244) of the procedures. The exclusion criteria were: subtotal or partial hysterectomy; hysterectomies associated with open rectosigmoidectomy and/or partial cystectomy due to endometriosis; and malignant diseases requiring total hysterectomy with open retroperitonial exploration.

Patient-specific (age, parity, previous cesarean sections, abdominal surgeries and endometriosis) and surgery-related (hospital stay, operative time, rate of conversion to open procedure, uterine volume, intra- and postoperative complications) variables were analyzed.

The postoperative complications were divided according to the Clavien-Dindo (C-D) classification (Table 1), which was created in 1992 (by Clavien PA, Dindo D and Demartines N at University Hospital of Zurich, Zurich, Switzerland) is widely used, and is based on the type of therapy needed to correct the complication.^21^

**Table 1:** Clavien-Dindo classification

Grades	Definition
Grade I	Any deviation from the normal postoperative course without the need for pharmacological treatment or surgical, endoscopic and radiological interventions. The allowed therapeutic regimens are: drugs as antiemetics, antipyretics, analgesics, diuretics and electrolytes and physiotherapy. This grade also includes wound infections opened at the bedside.
Grade II	Requiring pharmacological treatment with drugs other than those allowed for grade I complications. Blood transfusions and total parenteral nutrition are also included.
Grade III	Requiring surgical, endoscopic or radiological interventions.
IIIa	Intervention not under general anesthesia..
IIIb	Intervention under general anesthesia
Grade IV	Life-threatening complications (including central nervous system complications)* requiring management at intermediate care or intensive care unit.
IVa	Single-organ dysfunction (including dialysis).
IVb	Multiple-organ dysfunction.
Grade V	Death of a patient.

Note: *Brain hemorrhage, ischemic stroke, subarachnoid bleeding, but excluding transient ischemic attacks.

In our institution, we receive every year 4 first-year residents (PGY-4 OB/GYN) of the Gynecologic Endoscopy and Endometriosis Fellowship Program, and 2 second-year residents (PGY-5 OB/GYN) of the Gynecologic Endoscopy and Endometriosis Fellowship Program. The surgeries were divided into three different time-frame groups reflecting distinct benchmarks of the SLSST curriculum implemented for the Gynecology Endoscopy and Endometriosis Fellowship Program: 2008-09 (I-1) – TLH performed by senior attending physicians with more than 5 years of experience in laparoscopic surgery; 2010-11 (I-2) – TLH performed by the new first-year residents (PGY-4 OB/GYN) before the implementation of the SLSST, supervised by senior physicians; and 2012-14 (I-3) –TLH performed by the new first-year residents (PGY-4 OB/GYN) after the implementation of the SLSST. The surgeries performed during the first 14 weeks of the SLSST (dominant hand training period) were excluded from this group. The surgeries were assisted and supervised by a second-year (PGY-5 OB/GYN) resident who was also submitted to the same training program, but at the end of the previous year.

An average of 7 TLHs per PGY-4/year was observed in the I-2 group, and an average of 14 TLHs per PGY-4/year was observed in the I-3 group.

### Standard Total Laparoscopic Hysterectomy

The intrafascial technique has been standardized to all TLHs performed by the Gynecologic Endoscopy and Endometriosis Group of Santa Casa de Misericórdia de São Paulo since 2008. The same standard steps are performed in the same order for every TLH.

**Fig. 1:**
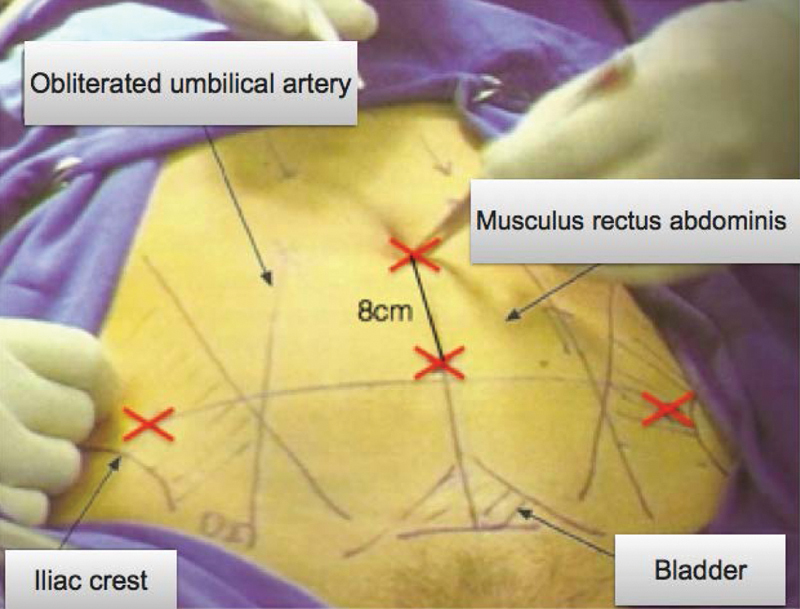
Locations of the portals.

**Fig. 2:**
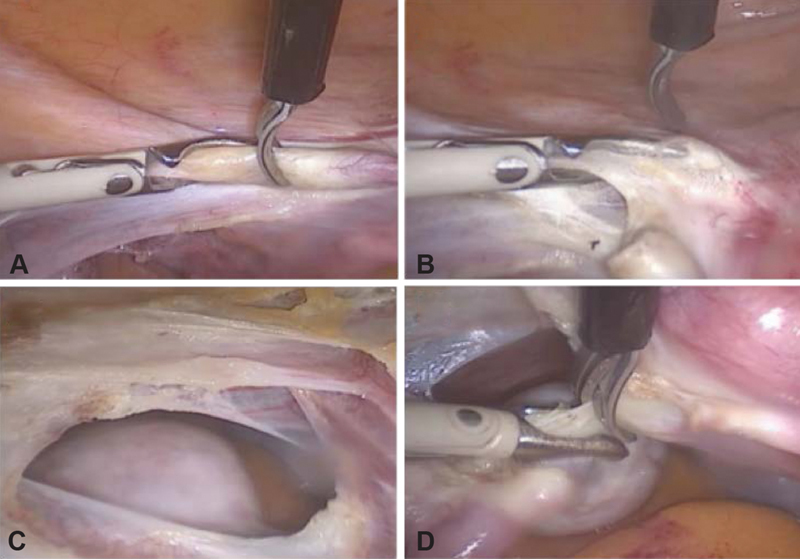
(**A**) Hemostatic section of the left round ligament; (**B**) dissection of the left broad ligament of the uterus; (**C**) left avascular window; (**D**) hemostatic section of the left uterus-ovarian ligament.

**Fig. 3:**
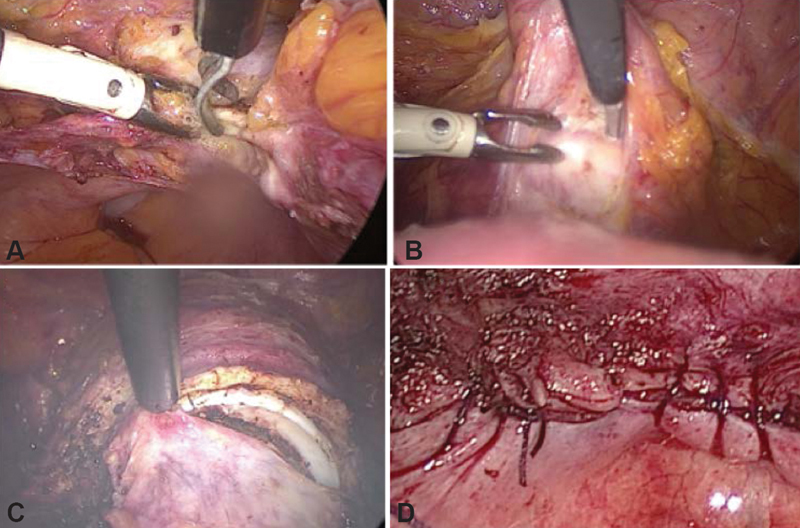
(**A**) Section of the left uterine vessels; (**B**) lower retraction of the bladder; (**C**) colpotomy; (**D**) colporrhaphy.

1. Position on the gynecological table with the legs on the gaiters, the buttocks 5 cm above the table, and arms on jambs along the body, asepsis/antisepsis, followed by late bladder catheterization using a no. 14 Foley catheter, hysterometry and uterine manipulator placement.
2. Intra-umbilical incision (longitudinal or arch-shaped), abdominal puncture using a Veress needle followed by safety maneuvers (dual recoil test, saline infusion-suction test, and pendant drop test), peritoneum distention with CO_2_ (6 mmHg to 20 mmHg), assessment of abdominal distension symmetry, and loss of liver solidness to percussion sign.
3. Introduction of the intra-umbilical trocar and cavity inventory to assess puncture accidents and adherences. Low pneumoperitoneum pressure to 14 mmHg, Trendelemburg position, and establishment of accessory portals using 5-mm trocars (Figure 1).
4. Left round ligament styptic section, dissection of the anterior peritoneum from the broad ligament to the bladder reflex, and establishment of an avascular plane left uterus-ovarian ligament styptic section, followed by left salpingectomy. If left oophorectomy is required, identify the left ureter and perform the styptic section of the infundibulum (Figure 2).
5. Dissect the posterior peritoneum from the broad ligament of the uterus to the sacrouterine ligament and perform the styptic section of the left uterine vessels (Figure 3A).
6. The same sequence (4 and 5) is then repeated on the right side.
7. Bladder retracted inferiorly (Figure 3B), colpotomy using a monopolar cautery (Figure 3C), and transvaginal removal of the uterus employing a vaginal liner.
8. Place a vaginal tampon to hold the pneumoperitoneum, followed by trans-peritoneal colporrhaphy using no. 0 Vicryl (polyglactin 910 manufactured by Ethicon Inc., a subsidiary of Johson and Johnson) with x-shaped stitches at the angles of the vagina and continuous stitches in the center (Figure 3D).
9. Review the hemostasis, remove the vaginal tampon, perform the suction of the pneumoperitoneum, return to horizontal decubitus, and perform the intra-umbilical aponeurosis suture and trocar incision closure.

### Systematic laparoscopic skills and suture training (SLSST)

The training was implemented to the curriculum of the Gynecologic Endoscopy and Endometriosis Fellowship Program in 2012. Based on the Romeo Gladiator Rule seven-week activities^22^ (Table 2), the SLSST consisted of a 21-week (4 hours per week) hands-on training at the experimental laboratory. Each week, the residents had to practice the scheduled exercise for a minimum of 4 hours. The core exercises were performed with the dominant hand on the lateral trocar for the initial 7 weeks of the program, followed by the dominant hand on the central trocar from the 8th to the 14th weeks, and the non-dominant hand on the lateral trocar from the 15th to the 21st weeks.

**Table 2:** Systematic laparoscopic skills and suture training core program

Week	Exercises
1st	Gladiator navigation
2nd	Gladiator with knot technique
3rd	Needle and guidelines
4th	Check-cross, deep and shallow stitches (staggering)
5th	Simple stitch with knot (number and resistance)
6th	Vaginal apex: x-shaped stitches, continuous suture right to left and left to right
7th	Myomectomy sutures and invaginating stitches
**1st to 7th:** Dominant hand on lateral trocar **8th to 14th:** Dominant hand on central trocar* 15th to 21st: Non-dominant hand on lateral trocar*	

Notes: Training based on the Romeo Gladiator Rule.^22^ *Same exercise sequence as weeks 1 to 7.

### Statistical Analysis

All data were recorded in Excel (Microsoft Corp., Redmond, WA, US), version 14.5.7, spreadsheets, and the statistical analyses were performed using in the Statistical Package for the Social Sciences (SPSS, IBM Corp., Armonk, NY, US) software, version 22. The calculation of the sample size was estimated based on a pilot group of 10 patients obtained from the first period of time (I-1). Using a standard deviation of 50 minutes and an estimated difference of 30 minutes, a sample of 34 patients was suggested to obtain a study power of 80% with a significance of 5%. The Kolmogorov-Smirnov test was performed for each independent variable to determine normal distribution. The Chi-squared test was used to compare parametric variables. The Student *t*-test was used to compare parametric continuous variables, and logistic regression was performed to determine the association between the variables. Data are shown as mean ± standard deviation (SD). Values of *p *< 0.05 were considered significant for the inferential analysis.

## Results

We included 244 cases of TLH in the study: 24 operated in 2008-09 (I-1); 55 in 2010-11 (I-2); and 165 in 2012-14 (I-3). The increase in TLHs performed per year at our hospital between periods I-1 and I-2 was of 129%, and between I-2 and I-3, it was of 100%. The mean age of the patients was 45,93 ± 8,37 (SD) years. Patient-specific variables are reported on table 3. The procedures performed together with TLH, like salpingectomy, oophoroplasty/oophorectomy, and deep endometriosis are shown in table 3.

**Table 3:** Patient-specific variables and procedures performed

Features		n	%
Parity	0	22	9.0%
	1	29	11.9%
	≥2	193	79.1%
Previous cesarean section		117	48.0%
Endometriosis		44	18.0%
Previous abdominal surgeries (including cesarean section)		184	75.4%
Total laparoscopic hysterectomy only		102	41.8%
*plus salpingectomy*		76	31.1%
*plus ovarian surgery*		35	14.3%
*plus endometriosis*		24	9.8%
*plus others procedures*		07	2.9%

The clinical indications for TLH were mainly uterine myoma (66.4%) and endometriosis (16.4%). Only two cases were diagnosed with malignant disease, and they were referred to the gynecologic oncology service after surgery. Comparing the three groups studied, we observed a significant difference in the number of previous cesarean sections, previous abdominal surgeries, rate of conversion to open surgery, and hospital stay longer than 2 days (Table 4). Out of 244 TLHs, 3 were converted to open laparotomy due to high uterine volume, and 1 required an open vaginal route. The overall complication rate was of 5.7%: 2.0% intraoperative and 3.7% postoperative complications.

**Table 4:** Clinical indications and associated variables stratified by time period

	2008-09 (I-1)	2010-11 (I-2)	2012-14 (I-3)	*p*-value
Myomatosis	70.8%	56.4%	73.5%	0.52
Endometriosis	12.5%	20.0%	18.0%	0.72
Previous cesarean section	37.5%	32.7%	54.5%	0.02
Comorbidities	50.0%	56.3%	55.1%	0.86
Previous abdominal surgeries	45.8%	60.0%	69.7%	0.03
Conversion	4.1%	1.8%	1.2%	0.04
Hospital stay > 2 days	29.1%	49%	18.1%	0.01
Intraoperative complications	0.0%	3.6%	2.4%	0.52
Postoperative complications	8.3%	3.6%	4.2%	0.21
Clavien-Dindo^21^–I	0.0%	0.0%	0.0%	
Clavien-Dindo – II	0.0%	0.0%	2,4%	
Clavien-Dindo – IIIa	0.0%	0.0%	0,6%	
Clavien-Dindo – IIIb	8,3%	1,8%	1,2%	
Clavien-Dindo – IVa	0.0%	1,8%	0.0%	
Clavien-Dindo – IVb	0.0%	0.0%	0.0%	
Clavien-Dindo – V	0.0%	0.0%	0.0%	

Note: Chi-squared statistical significance (*p* < 0.05).

We observed 6 intraoperative complications (1 internal iliac artery lesion, 1 acute respiratory failure, 2 sutured bladder lesions and 2 vaginal wall lacerations) and 11 postoperative complications (1 left iliac fossa seroma – C-D II; 1 umbilical hernia – C-D IIIb; 1 wall endometrioma – C-D IIIb; 2 vaginal dome granulomas – C-D IIIb; 1 buckling in the distal left ureter with loss renal function – C-D Iva; 1 intraperitoneal vesical fistula – C-D IIIa; 1 vaginal dome bleeding – C-D IIIb; 1 urinary retention – C-D II; 1 paralytic ileus – C-D II; and 1 postlumbar-puncture headache – C-D II).

We observed a decrease in uterine volume in group I-2 compared to group I-1: 217.8 cm^3^ and 154.2 cm^3^ respectively (*p* = 0.03). There was no difference in the uterine volume between groups I-2/I-3 and I-1/I-3. The operative time was longer in group I-2 when compared to group I-3 (p = 0.01); there was no difference between groups I-1/I-2 and I-1/I-3 (Table 5). The logistic regression showed an association between uterine volume and baseline uterus disease (*p* = 0.02), comorbidities (*p* = 0.03), and type of surgery performed (*p* < 0.001). The operative time showed association with baseline uterus disease (*p* = 0.001), hospital stay > 2 days (*p* = 0.002), endometriosis (*p* = 0.002), and intraoperative complications (*p* = 0.013). No significant association was found between uterine volume and operative time (*p* = 0.10) (Table 6).

**Table 5:** Uterine volume and operative time by group

	2008-09 (I-1)	2010-11 I-2	2012-14 I-3
**Uterine volume (cm^3^;** mean ± standard deviation**)**	217,8 ± 159,5	154,2 ± 95,9*	180,8 ± 91,4
**Operative time (min;** mean ± standard deviation**)**	219,8 ± 50,0	228,8 ± 89,1	204,5 ± 51,9**

Notes: *t*-test statistical significance (*p* < 0.05); *I-1 versus I-2: *p* = 0.03; **I-2 versus I-3: *p* = 0.01.

**Table 6:** Logistic regression results

Associations	*p*-value
Uterine volume (cm^3^) x baseline uterus disease	*p* = 0.02
Comorbidities	*p* = 0.03
Type of surgery performed	*p* < 0.001
Operative time (min) x baseline uterus disease	*p* = 0.001
Hospital stay > 2days	*p* = 0.002
Endometriosis	*p* = 0.002
Intraoperative complications	*p* = 0.013
Uterine volume (cm^3^) x operative time (min)	*p* = 0.10

Note: Statistical significance (*p* < 0.05).

## Discussion

The data confirmed our hypothesis that the SLSST would have a significant impact on TLH outcomes. The procedures performed by the SLSST-trained residents (group I-3) presented a reduction in operative time, length of hospital stay and conversion, reinforcing that a systematic training program can shorten the long learning curve, improve performance, and promote safe laparoscopic surgical practice in a teaching hospital.^23^ Technique standardization for TLH contributed to make the surgical outcomes comparable regardless of the surgeon who performed the procedure, and made the training process easier for the residents, who were no longer exposed to multiple technique variations.

We observed an increasing number of TLHs during the time-frames analyzed in the present study. The mean annual number of procedures more than tripled after the implementation of the training program. The complexity of the surgeries also increased, with bilateral salpingectomy becoming routine in 2013, but it did not increase the surgical time, hospital stay, or the complication rates. The bilateral salpingectomy became routine for all TLHs in order to decrease the risk of ovarian cancer.^24^ These findings may be related to an increased confidence and proficiency in performing more complex laparoscopic procedures after the training program. When analyzing the hospital stay for patients submitted to TLH, one should consider quantitative data or a qualitative approach. Considering that the majority of the patients were discharged between the first and second postoperative days, we preferred to use qualitative data and a cutoff of two days of hospitalization. The length of our hospital stay was consistent with the current literature, and the rate of complications was half of those reported in the literature.^25^ We found higher prevalence rates of previous cesarean sections (48%) and endometriosis (18%) than those reported in the literature,^23^ ^26^ which may be related to the extraordinary number of cesarean sections performed in Brazil,^25^ and to the fact that our hospital is a center of excellence for endometriosis care.

The present study did not find an association between uterine volume and operative time, neither between uterine volume and rate of complications. Our trained residents were able to significantly reduce TLH operative time in about 25 minutes, despite the fact that they resected a higher volume of uteri than the non-trained residents, suggesting that adequate training provided time-efficient abilities to young surgeons.

The traditional apprentice-tutor model is no longer valid to develop all skills necessary in gynecological surgery; the complexity of modern surgery has increased the demands and challenges to surgical education and the quality control.^27^ Simulators motivate residents through the journey of proficiency in laparoscopy.^28^ The positive impact that simulator-acquired skills have on real surgeries was published in a recent systematic review.^19^ A positive relationship between systematic training in simulators and reduced operative time and complications were also reported in cases of bariatric and urologic laparoscopic surgery.^29^ ^30^ In a Turkish study, Asoglu et al^31^ concluded that a simulator lab improves the outcomes of hysterectomy performed at a teaching institution, and may play an adjunct role in developing the resident's surgical skills; the results found by them are in line with the findings of our study.

In the present study, repetitive practice in simulators enabled the fellows to improve their psychomotor skills without the fear of making mistakes that could had been fatal in an actual surgery. The mistakes were analyzed by tutors who provided feedback and guided the residents to overcome obstacles. Tutorship in a stress-free environment translated into faster, safer and efficient surgical performance even for more experienced attending physicians.^32^ Many countries have established systematic training on simulators as requirements for laparoscopic surgeons.^33^ The present study was the initial step to validate a laparoscopic training program in Brazil. Our model may stimulate other academic hospitals to expand their proficiency laparoscopic skills, serving a bridge to a safe and effective full practice of *in vivo* laparoscopy.

Our study had several limitations. The cross-sectional design did not enable us to establish a temporal relationship between the training and surgical outcomes, or to determine if the experience of the surgeon measured by the number of TLHs previously performed had any influence over the surgical outcomes. Moreover, the substantial difference in the number of TLHs among the groups, and the variable skill levels of the senior attending physicians whose surgeries were included in group I-1 may be possible biases. Further prospective studies may define the number of previous TLHs necessary to reduce complications. On the other hand, the present study was, to our knowledge, this first clinical study to apply and evaluate the interference of the Romeo Gladiator training method on the outcomes of a surgical procedure.

## Conclusion

The operative time for TLH was significantly shorter in the group of patients operated after the SLSST was introduced in our hospital.
